# Regional assessment of articular cartilage gene expression and small proteoglycan metabolism in an animal model of osteoarthritis

**DOI:** 10.1186/ar1756

**Published:** 2005-05-12

**Authors:** Allan A Young, Margaret M Smith, Susan M Smith, Martin A Cake, Peter Ghosh, Richard A Read, James Melrose, David H Sonnabend, Peter J Roughley, Christopher B Little

**Affiliations:** 1 Raymond Purves Research Laboratory, Institute of Bone and Joint Research, Royal North Shore Hospital, University of Sydney, St Leonards, New South Wales, Australia; 2 School of Veterinary and Biomedical Sciences, Murdoch University, Perth, Western Australia, Australia; 3 Genetics Unit, Shriners Hospital for Children, Montreal, Quebec, Canada

## Abstract

Osteoarthritis (OA), the commonest form of arthritis and a major cause of morbidity, is characterized by progressive degeneration of the articular cartilage. Along with increased production and activation of degradative enzymes, altered synthesis of cartilage matrix molecules and growth factors by resident chondrocytes is believed to play a central role in this pathological process. We used an ovine meniscectomy model of OA to evaluate changes in chondrocyte expression of types I, II and III collagen; aggrecan; the small leucine-rich proteoglycans (SLRPs) biglycan, decorin, lumican and fibromodulin; transforming growth factor-β; and connective tissue growth factor. Changes were evaluated separately in the medial and lateral tibial plateaux, and were confirmed for selected molecules using immunohistochemistry and Western blotting. Significant changes in mRNA levels were confined to the lateral compartment, where active cartilage degeneration was observed. In this region there was significant upregulation in expession of types I, II and III collagen, aggrecan, biglycan and lumican, concomitant with downregulation of decorin and connective tissue growth factor. The increases in type I and III collagen mRNA were accompanied by increased immunostaining for these proteins in cartilage. The upregulated lumican expression in degenerative cartilage was associated with increased lumican core protein deficient in keratan sulphate side-chains. Furthermore, there was evidence of significant fragmentation of SLRPs in both normal and arthritic tissue, with specific catabolites of biglycan and fibromodulin identified only in the cartilage from meniscectomized joints. This study highlights the focal nature of the degenerative changes that occur in OA cartilage and suggests that altered synthesis and proteolysis of SLRPs may play an important role in cartilage destruction in arthritis.

## Introduction

Articular cartilage exhibits unique hydrodynamic and viscoelastic properties that are largely attributable to its extracellular matrix (ECM), which equips diarthrodial joints with their weight-bearing properties and near frictionless articulation. Cartilage ECM is composed of a collagen network, predominantly type II, in which large chondroitin sulphate and keratan sulphate (KS) substituted proteoglycans (aggrecan) are entrapped. The negatively charged aggrecan glycosaminoglycan side-chains act to create an osmotic swelling pressure in the cartilage matrix that is resisted by tension developed in the collagen network [1]. The generation of a hydrostatic pressure within cartilage allows it to counteract the loads transmitted to it from the long bones during normal joint articulation.

The ECM of cartilage also contains the small leucine-rich proteoglycans (SLRPs) biglycan, decorin, fibromodulin and lumican, which have diverse functions as modulators of tissue organization, cellular proliferation, adhesion and responses to growth factors and cytokines [2,3]. The SLRPs all bind to fibrillar type I and/or II collagens [4-6] and, in the case of decorin, to fibromodulin and lumican; these interactions modulate the rate and ultimate diameter of collagen fibrils formed *in vitro *[7-9]. Decorin, biglycan and fibromodulin can also form complexes with transforming growth factor (TGF)-β and modulate the action of this growth factor [10,11]. The physical presence of the SLRPs, in addition to the minor type IX and XI collagens, on the surface of type II collagen fibrils has been proposed to restrict sterically the access of collagenases to sites of cleavage on the collagen fibrils [12]. Complexes of matrilin-1 and decorin or biglycan have also been reported to connect type VI collagen to aggrecan and type II collagen, further stabilizing the cartilage ECM [13]. It is evident that there is a complex interplay between the collagenous and proteoglycan components of the cartilage ECM that produces a biocomposite material with unique mechanical properties. Disruption of the normal balance of ECM components through altered synthesis or degradation will have important ramifications for the load-bearing capacity of cartilage.

Chondrocytes, the highly differentiated cells of cartilage, are responsible for maintaining a homeostatic balance between production and degradation of cartilage ECM [14,15]. The metabolic status of the chondrocyte is central to our understanding of the initiation and progression of osteoarthrits (OA) [16]. An initial anabolic response of chondrocytes in OA includes an upregulation of mRNA levels for the major structural components type II collagen and aggrecan, with an associated elevation in synthesis [17,18]. Degradation of the ECM is also elevated in these early stages in OA. Eventually, the biosynthetic machinery of the chondrocyte is unable to keep up with the anabolic demands and a net depletion of ECM occurs during the later stages of OA. Loss of key functional components combined with a disrupted architecture result in compromised tissue function, cell death and, eventually, cartilage loss down to subchondral bone.

Changes in SLRP metabolism in human OA are relatively poorly characterized, with both increased synthesis and degradation of individual molecules reported in arthritic human cartilage [19,20]. Their function within the collagen network means that changes in their tissue content may significantly alter the biomechanical integrity of cartilage. However, because SLRPs are also regulators of growth factor activity, changes in their synthesis and degradation may have significant effects on chondrocyte metabolism. It is unclear whether the changes in SLRP metabolism are restricted to the cartilage undergoing OA degeneration or are more generalized within arthritic joints. An understanding of the changes that occur with the onset and progression of cartilage degeneration in OA may provide important insights into potential regulatory steps in this process.

Animal models of OA have permitted longitudinal evaluation of spatial and temporal changes in joint tissues that occur during the development of joint disease. Total or partial removal of knee joint meniscus in humans commonly results in degeneration of articular cartilage, leading to osteoarthritic changes [21]. In sheep, lateral meniscectomy has been shown to reliably reproduce biochemical, biomechanical and histopathological alterations typical of OA [22,23]. In the present study we used this established model of OA to study the changes in expression of key structural molecules (aggrecan and type II collagen), the collagen-associated SLRPs (biglycan, decorin, lumican and fibromodulin), TGF-β_1 _and its associated downstream signaling molecule connective tissue growth factor (CTGF), and markers of altered chondrocyte phenotype – types I and III collagen. The expression levels were compared with protein levels in cartilage extracts or by immunohistochemistry in tissues with various histopathological grades of OA in the medial and lateral joint compartments.

## Materials and methods

### Animal model

Twelve 7-year-old female pure-bred Merino sheep were used in the present study. Six of the sheep underwent open lateral meniscectomy of both stifle joints, as previously described [24], whereas the remaining six served as nonoperated controls. Following recovery from surgery, the animals were maintained in an open paddock for 6 months before sacrifice. The protocol used for the present study was approved by the animal ethics committee of Murdoch University, Western Australia (AEC 832R/00).

### Tissue preparation

Full depth articular cartilage from the medial tibial plateau (MTP) and lateral tibial plateau (LTP) was sampled from either the right or left stifle (knee) joint, randomly selected. Care was taken not to sample tissue from the joint margins or osteophytes. Tissue samples were snap frozen in liquid nitrogen before storage at -80°C until they were required. The tibial plateaux from the contralateral joints were isolated by a horizontal cut through the tibia below the epiphyseal growth plate using a band saw. Full thickness coronal osteochondral slabs (5 mm) were subsequently prepared through the mid weight-bearing region of the tibial plateau.

### Histology

The coronal tibial osteochondral slices were fixed in 10% (vol/vol) neutral buffered formalin for 48 hours then decalcified in 10% formic acid (vol/vol)/5% formalin (vol/vol) for 5 days. The specimens were then dehydrated in graded alcohols and double-embedded in celloidin–paraffin blocks. Tissue sections (4 μm) were cut using a rotary microtome and attached to microscope slides. They were then deparaffinized in xylene and washed in graded alcohols to 70% (vol/vol) ethanol and then stained for 10 min with 0.04% (weight/vol) toluidine blue in 0.1 mol/l sodium acetate buffer (pH 4.0) to visualize the tissue proteoglycans. This was followed by 2 min counter-staining in an aqueous 0.1% (weight/vol) Food Drug and Cosmetic Green Nos. 3 stain. The slides were subsequently evaluated by bright field microscopy using a Leica MPS-60 (Leica Microsystems, Gladesville, New South Wales, Australia) photomicroscope system by two independent observers using a modified Mankin scoring scheme, previously developed in our laboratory for this ovine model [22]. In each compartment the worst score evident across the width of the tibial plateau was used to calculate the mean score for MTP and LTP of control and meniscectomized joints (*n *= 6 for each group).

### Immunohistochemistry

Immunostaining was performed using monoclonal antibodies against type I collagen (ICN Biomedicals, Aurora, USA; code no. 63170; clone no. I-8H5) and type II collagen (ICN Biomedicals, North Ryde, New South Wales, Australia; code no. 63171; clone no. II-4CII), and a polyclonal antibody against type III collagen (Cedarlane, Hornby, Ontario, Canada; code no. CL50321AP). Endogenous peroxidase activity was initially blocked by incubating the tissue sections in 3% (vol/vol) H_2_O_2 _for 5 min and the sections were rinsed in TBS-Tween.

For type I and II collagen localizations, the sections were predigested with proteinase K (Dako, Glostrup, Denmark; code no. S3020) for 6 min at room temperature, followed by bovine testicular hyaluronidase (Sigma, St Louis, MO, USA; code no. H-3506) 1000 U/ml for 1 hour at 37°C in phosphate buffer (pH 5.0). The type III collagen localizations were predigested with hyaluronidase alone. The sections were then incubated in 10% (vol/vol) swine serum for 10 min at room temperature to block any nonspecific binding.

Incubations with the primary antibodies were performed overnight at 4°C with type I (5 μg/ml), type II (10 μg/ml) and type III (1:500 dilution) collagens. Detection of primary antibody was undertaken using a 20 min incubation with a cocktail of biotinylated anti-rabbit and anti-mouse immunoglobulin secondary antibodies (Dako; code no. K1015), followed by a 20 min incubation with streptavidin-conjugated horseradish peroxidase (Dako; code no. K0690). Staining was undertaken using NovaRED substrate (Vector, Burlingame, CA, USA; code no. SK-4800) for 15 min, which gives a red–brown end product. Sections were counter-stained in Mayer's haematoxylin for 1 min, washed in H_2_O, dehydrated in ethanol, cleared in xylene and mounted. Negative control sections were prepared using irrelevant isotype matched primary antibodies (Dako; code no. X931 or X0936) in place of authentic primary antibody.

### RNA extraction

Approximately 100 mg of frozen cartilage samples was fragmented in a Mikro-Dismembrator (Braun Biotech International, Melsungen, Germany), 1 ml of TRIzol Reagent (Invitrogen Life Technologies, Carlsbad, CA, USA) was added, and the mixture was allowed to defrost to room temperature. Total RNA was isolated using the RNeasy Mini Kit from Qiagen (Valencia, CA, USA). Chloroform (300 μl) was subsequently added to the samples and the tubes vortexed vigorously before centrifugation to pellet the tissue residue. The clear supernatant solution (aqueous phase) was recovered and mixed by inversion with an equal volume of 70% ethanol, and then loaded onto spin columns. Following several washing steps and an on-column DNase digestion (Qiagen, Hilden, Germany), RNA was eluted from the column with 32 μl of RNAse free distilled H_2_O. Total RNA was quantified using a flourimeter (Perkin Elmer, Beaconsfield, UK) using SYBR^® ^Green II colour reagent (Cambrex Bio Science, Rockland, ME, USA), and each sample was assessed for purity to confirm the absence of detectable DNA.

### Semiquantitative RT-PCR

RT reactions were undertaken with 1 μg total RNA using the Omniscript RT kit from Qiagen (Germany). Using specific primer sets (Sigma Genosys, Castle Hill, New South Wales, Australia; Table 1), aliquots of cDNA were amplified by PCR, with initial denaturation at 94°C for 5 min, followed by cycles of 30 s of denaturation at 94°C, 30 s annealing at variable primer specific temperatures (Table 1), 30 s for extension at 72°C, and a further 7 min extension at 72°C on completion of the cycles. Reactions generated single PCR products that were identified by sequencing (SUPAMAC, Sydney, Australia) and specificity confirmed by BLAST searches. Cycle optimization was performed for each primer set before PCR, and for all reported experiments amplification levels were compared in the linear range of the PCR reaction. All samples underwent RT and cDNA amplification at the same time to avoid potential variations in experimental conditions.

The amplified products were electrophoresed on 2% (weight/vol) agarose gels, stained with ethidium bromide, imaged using a Fujifilm FLA-3000 fluorescent image analyzer and integrated densities calculated using One-Dscan, 1-D gel analysis software (Scanalytics, Fairfax, VA, USA). Sample loadings were normalized to the housekeeping gene GAPDH (glyceraldehyde-3-phosphate dehydrogenase) to permit semiquantitative comparisons in mRNA levels, as previously described [25,26].

### Cartilage extraction, SDS-PAGE and Western blotting of the small leucine-rich proteoglycans

Pooled cartilage samples from all meniscectomized and nonoperated control LTPs were finely diced and extracted with 10 volumes of 4 mol/l GuCl and 50 mmol/l Tris HCl (pH 7.2) in the presence of proteinase inhibitors at 4°C with end over end stirring for 48 hours before dialysis of the extract against ultrapure water, as described previously [27]. Insufficient cartilage was available from MTPs for extraction and Western blot analyses. Dialysed extracts corresponding to equal dry weights of tissue were predigested with either chondroitinase ABC (Seikagaku) 0.1 U/ml alone or in combination with keratanase II (Seikagaku) 0.01 U/ml and endo-β-galactosidase (Seikagaku, Tokyo, Japan) 0.01 U/ml in 0.1 mol/l Tris/0.1 mol/l sodium acetate (pH 7.0) overnight at 37°C before electrophoresis. Electrophoresis was conducted under reducing conditions in 10% NuPAGE Bis-Tris resolving gels (Invitrogen), using MOPS SDS running buffer at 125 V constant voltage for 1 hour. The gels were then electroblotted to nitrocellulose membranes in NuPAGE transfer buffer with 20% (vol/vol) methanol at 200 mA for 2 hours and blocked overnight in 5% (weight/vol) BSA in 50 mmol/l Tris-HCl (pH 7.2) and 0.15 mol/l NaCl 0.02% (weight/vol) NaN_3 _(TBS-azide). The blots were probed overnight with affinity purified polyclonal antibodies directed against the carboxyl-terminus of decorin, biglycan, fibromodulin and lumican (0.3–1 μg/ml) [12] followed by washing in TBS-azide and detection using alkaline phosphatase conjugated anti-rabbit secondary antibodies and the nitro blue tetrazolium/4-bromo-1-chloro-indolyl phosphate substrate system (BioRad, Hercules, CA, USA). A sample of human OA cartilage harvested from the tibial plateau at the time of joint replacement surgery also underwent identical processing as a positive control.

### Statistical analysis

All RT-PCR data were normalized to the housekeeping gene GAPDH (glyceraldehyde-3-phosphate dehydrogenase) to facilitate equal loading of gels for quantitative comparisons of amplified PCR products. Comparison of parametric data from the nonoperated and meniscectomized sample groups were undertaken using the unpaired Student's t-test with Benjamini–Hochberg correction [28] for multiple comparisons. Comparisons of nonparametric data from the modified Mankin histological scoring of the stained tissue sections were assessed using the Mann–Whitney U-test.

## Results

### Histology

**Figure 1:**
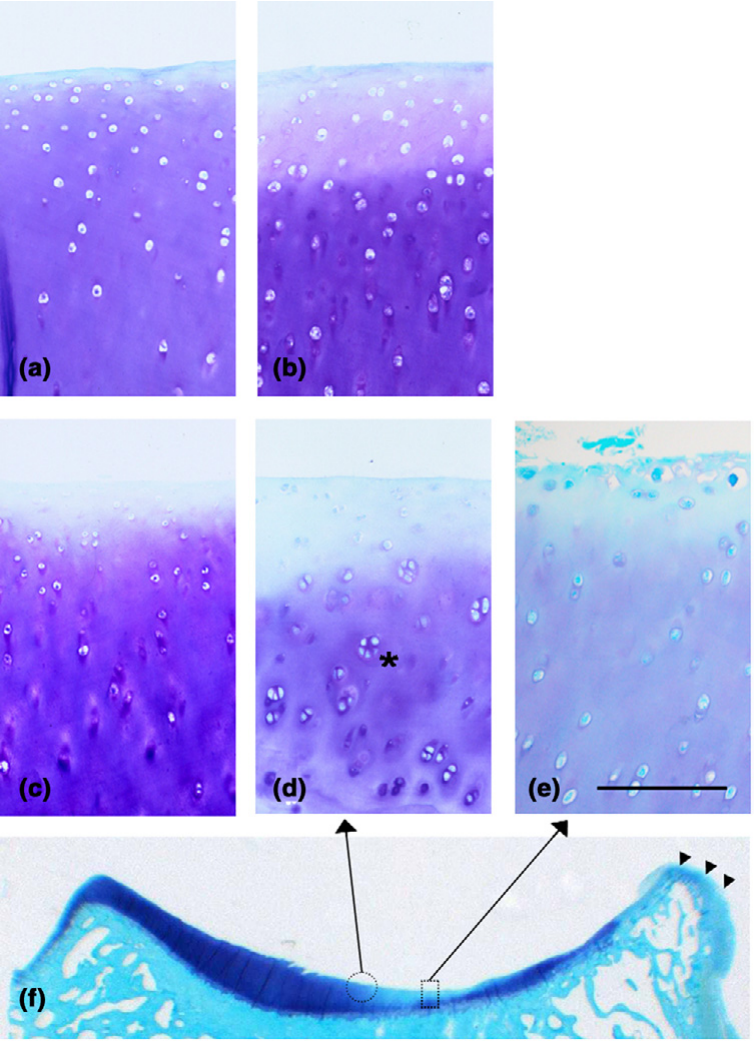
Histology. Representative histology of medial **(a, b) **and **(c–f) **lateral tibial plateau cartilage from nonoperated control (panels a and c) and meniscectomized (panels b and d-f) ovine stifle joints. Cell cloning is a prominent feature in the lateral tibial plateau following meniscectomy (asterisk). Osteophyte formation is evident at the lateral joint margin (panel f, arrowheads), and the area of most severe cartilage damage with surface fibrillation (rectangle, panel e) and the adjacent area (circle, panel d) are indicated. Toluidine blue-fast green stain. Scale bar: 250 μm.

Lateral meniscectomy resulted in macroscopic joint changes characteristic of the early and middle phases of OA with cartilage fibrillation and erosion, in addition to formation of marginal osteophytes, particularly in the lateral compartment (Fig. 1f; arrowheads). The histopathological lesions varied between animals, between medial and lateral joint compartments, and across the width of the tibial plateaux. A significant loss of proteoglycan was evident in the superficial cartilage of both the LTP (Fig. 1d, e) and, to a lesser extent, the MTP of the meniscectomized joints (Fig. 1b) compared with nonoperated controls (Fig. 1a, c). Chondrocyte cloning was also a prominent feature in the LTP specimens after meniscectomy (Fig. 1d; asterisk), which is in keeping with the validity of this model's representation of human OA. The most severe lesions were confined to the weight-bearing region of the LTP, with significant proteoglycan loss and surface fibrillation (Fig. 1e, f).

Histological grading of the meniscectomized and nonoperated control cartilage specimens confirmed and quantitated the histological observations. In control sheep the modified Mankin score (mean ± standard deviation) was significantly higher in the MTP specimens than in the LTP ones (9.3 ± 1.9 versus 3.1 ± 1.1; *P *< 0.01). Following meniscectomy there was a slight although not statistically significant change in the modified Mankin score for the MTP specimens (10.7 ± 3.3). The same could not be said of the LTP specimens, in which meniscectomy resulted in a significant increase from 3.1 ± 1.1 to 23.3 ± 1.8 (*P *< 0.01).

### Immunolocalization of types I, II and III collagens

**Figure 2:**
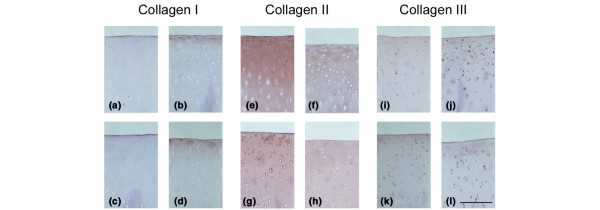
Immunolocalisation. Immunolocalization of types I **(a–d)**, II **(e–h) **and III **(I–l) **collagens in medial (panels a, b, e, f, I and j) and lateral (panels c, d, g, h, k and l) tibial plateau cartilage. Sections from representative nonoperated control (panels a, c, e, g, I and k) and meniscectomized (b, d, f, h, j and l) joints are shown. Scale bar: 250 μm.

An increase in type I collagen matrix immunostaining was evident following meniscectomy in the most superficial cartilage of the LTP specimens (Fig. 2d) and, to a lesser extent, in the MTP specimens (Fig. 2b), corresponding to areas of degenerative change. In nonoperated control sections (Fig. 2a, c), type I collagen was restricted to the uppermost surface lamina, as reported previously [29]. Type III collagen, which is typically seen pericellularly in normal cartilage [30], also exhibited increased matrix staining after meniscectomy (Fig. 2j, l) compared with nonoperated control (Fig. 2i, k). Type II collagen was immunolocalized in the matrix throughout the depth of the cartilage in both MTP and LTP, and there was a generalized decrease in staining following meniscectomy (Fig. 2e–h). As expected [31,32], types I and III collagens were also prominently immunolocalized in the marginal osteophytic fibrocartilaginous regions in the meniscectomized joints (data not shown).

### RT-PCR

**Figure 3:**
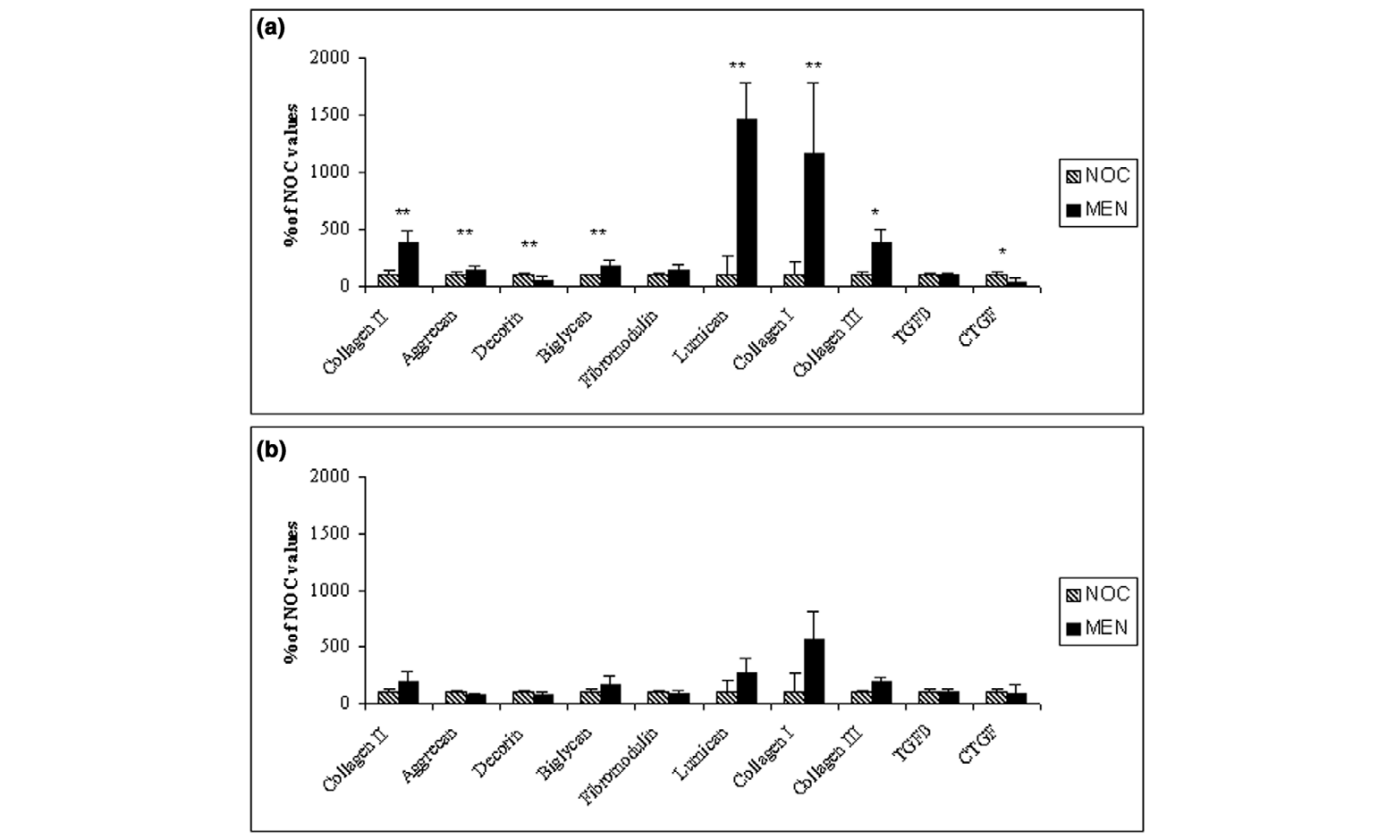
Changes in mRNA levels. Changes in **(a) **lateral tibial plateau (LTP) and **(b) **medial tibial plateau (MTP) cartilage mRNA levels of aggrecan, type I, II, and III collagen, decorin, biglycan, fibromodulin, lumican, transforming growth factor (TGF)-β and connective tissue growth factor (CTGF) following lateral meniscectomy (MEN) relative to nonoperated control (NOC) values. Values are expressed as mean ± standard deviation. There were three samples for the NOC MTP, six for the NOC LTP, five for the MEN MTP and six for the MEN LTP groups. *P < 0.05, **P < 0.01.

It was not possible to undertake all procedures with some of the cartilage samples that did not yield at least 1 μg total RNA. This resulted in four samples being excluded, all from MTP cartilage (one from the meniscectomy group and three from the nonoperated control group). Statistical comparisons of mRNA levels following meniscectomy as a percentage of control values were undertaken separately for LTP and MTP cartilages and are presented graphically in Fig. 3. Following lateral meniscectomy, mRNA levels in LTP cartilage were found to be upregulated for the following molecules: aggrecan (1.5 fold; *P *< 0.01), type I collagen (11.7-fold; *P *< 0.01), type II collagen (3.9-fold; *P *< 0.01), type III collagen (2.3-fold; *P *< 0.05), biglycan (1.8-fold; *P *< 0.01) and lumican (14.6-fold; *P *< 0.01). In the same region there were downregulations of decorin (1.6-fold; *P *< 0.01) and CTGF (2.1-fold; *P *< 0.05), and unchanged expression of fibromodulin and TGF-β. In the MTP cartilage samples, none of the changes in mRNA levels following meniscectomy relative to nonoperated controls were statistically significant.

### Western blotting of the small leucine-rich proteoglycans

**Figure 4:**
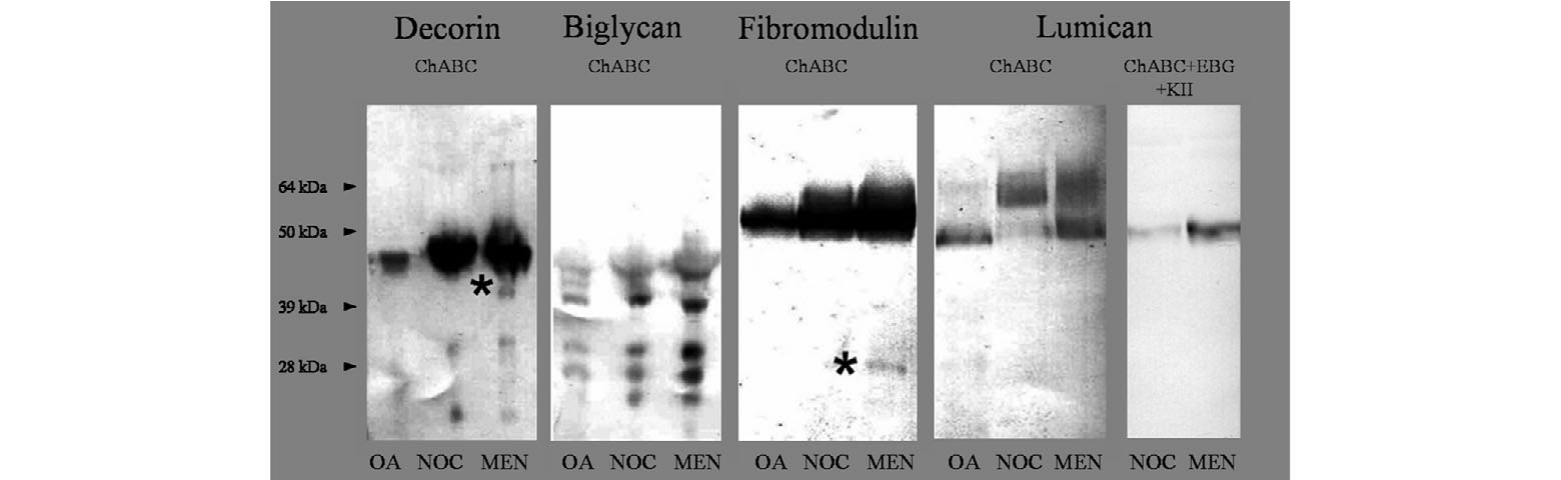
Western blot. Western blot analysis of decorin, biglycan, fibromodulin and lumican in extracts of human osteoarthritis cartilage (OA), nonoperated control (NOC) and lateral meniscectomized (MEN) ovine cartilage samples. Core protein fragments of decorin and fibromodulin that were only identified in MEN are marked with an asterisk. Equivalent amounts of extract from equal dry weights of tissue were loaded per lane following treatment with chondroitinase ABC (ChABC). Additionally, Western blot analysis of lumican was performed following treatment with ChABC, endo-β-galactosidase (EBG) and keratanase II (KII). The migration positions of prestained protein standards are indicated on the left.

Western blot analysis of extracts of an equivalent dry weight of pooled LTP cartilage from control and meniscectomized joints and OA human cartilage are shown in Fig. 4. There was little difference in total staining intensity between nonoperated and meniscectomized cartilage for the 45 kDa intact core protein of decorin that was also evident in human OA cartilage. Additional fragmented forms of decorin core protein (32 and 20 kDa) were evident in the cartilage extracts from both the control and meniscectomy specimens, whereas a 40 kDa fragment was identified only in meniscectomized cartilage extracts (Fig. 4; asterisk). Blotting for biglycan identified intact core protein (43 kDa) and a number of fragments (39, 32, 28 and 26 kDa) in all of the specimens. There was an increase in staining intensity for all biglycan core protein species in meniscectomized cartilage. The predominant fibromodulin core protein species identified in all specimens was about 55 kDa in size, with a slight increase in staining following meniscectomy. This 55 kDa fibromodulin band is consistent with full-length core protein [12]. A 28 kDa fibromodulin fragment was detected only in the extract from meniscectomized joints (Fig. 4; asterisk). Lumican electrophoresed as two predominant species, a 60–64 kDa band with similar staining intensities evident in control and meniscectomy extracts. A smaller, approximately 50 kDa band, which was the predominant species in the human OA sample, exhibited greater staining intensity after meniscectomy compared with cartilage from nonoperated joints. Removal of KS side-chains with keratanase II/endo-β-galactosidase treatment resulted in all of the lumican migrating at 50 kDa, suggesting that the 60–64 kDa band represented KS substituted lumican.

## Discussion

Our laboratory previously reported biochemical, biomechanical and histological changes that occur in the articular cartilage in the ovine lateral meniscectomy model of OA [22,23,33]. The present study extends these earlier investigations by examining the expression of a number of important extracellular matrix components at the mRNA level. One of the difficulties we encountered was relatively low average RNA yields (0.85–9.13 μg per 100 mg), which resulted in exclusion of some MTP samples. Studies utilizing other animal models of OA have reported RNA yields from 2.5 to 21 μg/100 mg of normal cartilage [34,35], but the animals used in those studies (rabbit and canine) were of a much younger age than ours. Studies using aged human cartilage report much lower average yields, ranging from 0.669 to 0.839 μg/100 mg of OA and 'normal' cartilage [36]. We attributed the low RNA yield in our study to our use of an aged population of sheep, although other factors such as species differences, RNA degradation and technical factors cannot be excluded. Although we were able to analyze medial and lateral tibial cartilage separately, the low yields of RNA from the older sheep precluded further topographical separation. Future studies using younger animals may permit analysis of affected and unaffected cartilage within one joint area.

Although morphological and histological changes in cartilage were most notable in the lateral compartment, changes in the medial femoro-tibial joint were nevertheless still evident but of a markedly lesser magnitude, as previously reported [23,37]. In the present study the MTP cartilage in control joints had significantly worse histopathological scores than did LTP from the same joints, which is consistent with age-related change in the more heavily loaded compartment of these old animals. The histopathology scores did not increase significantly in the medial compartment following meniscectomy, and this was consistent with the lack of change in mRNA levels. Our inability to detect differences in mRNA expression in the medial compartment might have resulted from the small number of samples evaluated. However, the standard deviation of the MTP samples was similar to that of the LTP, suggesting that the lower number of MTPs studied did not contribute to the lack of statistical significance. Changes in mRNA levels for a number of molecules were significant in the lateral compartment following meniscectomy. Although our findings are limited to a single time point following induction of OA, restriction of significant alterations in gene expression to the LTP indicates that the changes observed were likely associated with active degradation of cartilage primarily due to altered biomechanical forces rather than humoral factors.

In the present study the changes observed in the expression of aggrecan and type II collagen probably reflect an anabolic response by the chondrocytes to the altered mechanical stresses imposed by this surgical procedure, as well as early OA degeneration. The increase in expression is consistent with an attempted 'repair' response in early OA, as described in other animal models [34,35,38]. Levels of mRNA for a particular molecule may not reflect protein synthesis or its accumulation in tissue, with post-transcriptional regulation and post-translational processing playing significant roles. Indeed, we previously demonstrated increased degradation of newly synthesized aggrecan in cartilage after lateral meniscectomy in sheep [24]. Furthermore, the changes in mRNA levels observed in the present study were representative of the entire MTPs or LTPs and therefore probably included cartilage from areas with different stages of OA.

In addition to the increase in mRNA for the major cartilage matrix components aggrecan and type II collagen, significant increases in expression and protein levels of types I and III collagen were observed following meniscectomy. Type III collagen is present pericellularly in small amounts in normal articular cartilage [16,30], and type I collagen is is evident in the most superficial layer [29]. Contrary to early reports [39], evidence now suggests that both types I and III collagens are significantly increased in OA cartilage, both at the expression and protein levels [40,41]. It has been suggested that a major phenotypic shift occurs in OA toward a de-differentiated chondrocyte [40]. Interestingly, in the present study we observed increased amounts of types I and III collagens by immunohistology in both compartments following meniscectomy, despite increased mRNA levels only being evident in the lateral compartment. A probable explanation was that the increased types I and III collagens observed with immunohistochemistry represented the cumulative changes throughout the course of the disease process while expression levels reflected chondrocyte metabolism at a specific point in time (i.e. 6 months following meniscectomy). Changes in collagen subtypes in pathological cartilage may not only influence the biomechanical integrity of the tissue but may sequester and modulate the actions of cytokines, with types I and III collagen shown to bind oncostatin M specifically [42].

Selective modulation of SLRP mRNA levels in OA cartilage was observed in the present study, with increased biglycan and lumican, decreased decorin, and little or no change in fibromodulin. Additionally, we have shown for the first time that these changes in SLRP expression are confined to the cartilage in the compartment undergoing active OA degeneration. The differential regulation contrasts with the reported increase in expression of all four SLRPs in late-stage human OA in one study [19], but it is consistent with another study [43] that reported no change in decorin but increased biglycan message in late stage OA. In the canine anterior cruciate ligament transection model, increased cartilage mRNA for biglycan, decorin and fibromodulin have been described [38,44]. The reported differences in mRNA expression may relate to variable stages of disease, methods of quantitation and species evaluated.

The SLRPs have been shown to influence cartilage metabolism indirectly via actions on growth factors such as TGF-β, which they inactivate through sequestration and thereby potentially mitigate its effects in OA [11,45]. Although we found no change in the expression of TGF-β following meniscectomy, there was a significant decrease in mRNA levels of CTGF. We speculate that sequestration of TGF-β by the SLRPs may have accounted for the decrease in CTGF expression. Our results contrast with human cartilage, in which an increase in CTGF in OA was recently reported [46], and this could be associated with species differences or the stage of disease. CTGF, a secretory protein involved in fibrotic response mechanisms in tissues, is an important downstream effector of TGF-β [47] and is thought to be involved in promoting the proliferation and/or differentiation of chondrocytes [48-51]. Further investigation of the specific relationships between growth factors, collagens and the SLRPs in normal and diseased cartilage is warranted.

Significant proteolysis of the SLRPs was evident in the present study. SLRP degradation was previously reported in both human OA [20] and spontaneous canine OA [52], but not in a canine cruciate ligament transection model of OA [52]. The catabolites that were identified in meniscectomized cartilage in the present study were also generally evident in normal cartilage, indicating similar proteolytic processes in health and disease. However, in the case of decorin and fibromodulin, fragments unique to the meniscectomized cartilage were identified, suggesting the presence of disease-specific proteolytic processes. In this regard, a specific proteolytic fragment of fibromodulin was recently identified from interleukin-1 stimulated but not normal cartilage [53]. The cleavage site(s) and proteinase(s) responsible for extracellular SLRP breakdown in arthritic cartilage have yet to be identified and are the subject of further investigation.

A particularly novel finding in the present study was the increased lumican core protein present in degenerative cartilage following meniscectomy, which is consistent with the significant increase observed in mRNA levels. Furthermore, the increased lumican observed by Western blotting was present in a non-KS substituted form. Limited studies [19,54] have suggested that lumican primarily exists lacking KS in adult cartilage, but cultured chondrocytes have been observed to produce a KS-substituted form that appeared to be the default synthesis preference [55]. The catabolic cytokine interleukin-1β, which may be present in OA joints, stimulates secretion of lumican deficient in KS [55]. It has been shown that OA chondrocytes synthesize SLRPs that are differently glycosylated, and that nonglycosylated biglycan and decorin are more abundant in OA cartilage [20]. Changes in glycosylation of the SLRPs, whether by altered synthesis or subsequent degradation, are likely to influence the functional properties of these molecules in cartilage.

## Conclusion

We showed that degradation of cartilage in OA is associated with significant focal changes in expression and content of matrix proteins. Accelerated proteolysis of aggrecan and type II collagen overwhelms the increase in expression of these major structural proteins. Furthermore, there is a shift in chondrocyte phenotype, with increased synthesis of collagens types I and III and a change in the relative levels of the fibril-associated SLRPs. In particular there is decrease in synthesis of decorin and an increase in biglycan and lumican, with the latter lacking KS substitution. It seems likely that the altered pattern of SLRP synthesis, which is localized to the diseased joint compartment, along with an increase in SLRP proteolysis, modifies the biomechanical properties of the matrix and contributes to cartilage breakdown. Changes in SLRP levels could also significantly modulate the action of potential anabolic factors such as TGF-β and its downstream effector CTGF, possibly adding to disease development. An understanding of the relationship between SLRP metabolism and progressive cartilage breakdown in OA may provide both novel diagnostic markers of disease and therapeutic targets for the treatment of this disorder.

## Abbreviations

CTGF = connective tissue growth factor; ECM = extracellular matrix; KS = keratan sulphate; LTP = lateral tibial plateau; MTP = medial tibial plateau; OA = osteoarthritis; RT-PCR = reverse transcription polymerase chain reaction; SLRP = small leucine-rich proteoglycan; TGF = transforming growth factor.

## Competing interests

The author(s) declare that they have no competing interests.

## Authors' contributions

AAY conducted the RT-PCR and Western blotting studies and drafted the manuscript. MMS designed primers for RT-PCR, performed histopathological cartilage scoring and helped to draft the manuscript. SMS performed histological and immunohistological preparations, and helped to draft the manuscript. MAC performed animal surgery and helped to draft the manuscript. RAR performed animal surgery and helped to draft the manuscript. PG made substantial contributions to the conception and design of the study. JM assisted with performing Western blotting studies and helped to draft the manuscript. DHS was involved in the conception and design of the study, and interpretation of the data, and critically revised the manuscript for important intellectual content. PJR assisted with Western blotting studies and critically revised the manuscript for important intellectual content. CBL performed histopathological cartilage scoring, analyzed and interpreted the data, and critically revised the manuscript for important intellectual content. All authors read and approved the final manuscript.

## Figures and Tables

**Table 1:** Primers used for RT-PCR

Gene	Annealing temperature (°C)	Product size (base pairs)	Sequence (5' to 3')	GenBank accession number
Collagen II	65	141	F ACGGTGGACGAGGTCTGACT R GGCCTGTCTCTCCACGTTCA	AF138883
Aggrecan	65	375	F CCGCTATGACGCCATCTGCT R TGCACGACGAGGTCCTCACT	AF019758
Decorin	55	319	F CAAACTCTTTTGCTTGGGCT R CACTGGACAACTCGCAGATG	AF125041
Biglycan	65	204	F CCATGCTGAACGATGAGGAA R CATTATTCTGCAGGTCCAGC	AF034842
Fibromodulin	65	442	F CTGGACCACAACAACCTGAC R GGATCTTCTGCAGCTGGTTG	AF020291
Lumican	65	284	F CAGCCATGTACTGCGATGAG R CTGCAGGTCCACCAGAGATT	NM173934
TGF-β	60	271	F CGGCAGCTGTACATTGACTT R AGCGCACGATCATGTTGGAC	AF000133
CTGF	65	504	F TCTTCTGCGACTTCGGCTCC R CCTCCAGGTCAGCTTCGCAA	NM174030
Collagen I	65	460	F CCACCAGTCACCTGCGTACA R GGAGACCACGAGGACCAGAA	AF129287
Collagen III	55	243	F GCTGGCTAGTTGTCGCTCTG R GTGGGGAAACTGCACAACAT	L47641
GAPDH	55	320	F TCACCATCTTCCAGGAGCGA R GGCGTGGACAGTGGTCATAA	AF035421

Shown are the details of the primers used for RT-PCR, including annealing temperatures, size of the amplified products, forward (F) and reverse (R) sequences, and primer source. CTCG, connective tissue growth factor; GAPDH, glyceraldehyde-3-phosphate dehydrogenase; TGF, transforming growth factor.
